# Social prescribing in Canada: health promotion in action, 50 years after the Lalonde report

**DOI:** 10.24095/hpcdp.44.6.01

**Published:** 2024-06

**Authors:** Kate Mulligan, Kiffer G. Card, Sandra Allison

**Affiliations:** 1 Dalla Lana School of Public Health, University of Toronto, Toronto, Ontario, Canada; 2 Faculty of Health Sciences, Simon Fraser University, Vancouver, British Columbia, Canada; 3 University of British Columbia, Vancouver, British Columbia, Canada

**Keywords:** social prescribing, health promotion, health systems, population health

## Abstract

The Lalonde report, published in 1974 by the Canadian Minister of National Health and Welfare, broke ground for public health in Canada by acknowledging that the determinants of health are much broader than health care services. Fifty years later, this special issue of *Health Promotion and Chronic Disease Prevention in Canada* charts a clear path towards addressing upstream determinants of health through an emerging intervention called “social prescribing.” Social prescribing connects patients with community resources tailored to their individual priorities, fostering a paradigm shift from a deficit-based to a strengths-based approach in health promotion. Part 1 of this issue covers the rapid growth and diverse applications of social prescribing across Canada, with targeted initiatives for various populations and interventions ranging from nature and arts to physical activity and social connectivity. Contributions from a wide range of partners, including researchers, health professionals and community members, explore the adaptability of social prescribing for different groups, underscore the role of community and lived experiences in research, and call for more studies on social prescribing’s effectiveness and outcomes. Highlighted case studies demonstrate tangible benefits in health equity and access to social services. This issue not only reflects the current scope and impact of social prescribing in Canada but also sets the stage for its future development and integration into broader health practices.

## Introduction

This special issue of *Health Promotion and Chronic Disease Prevention in Canada* marks a national first—the first journal issue entirely dedicated to social prescribing, a rapidly growing field in Canada. The issue arrives 50 years after the 1974 Lalonde report highlighted the impact of social factors such as poverty and social isolation on health.^1^ Over the past half century, health care professionals and communities have increasingly recognized the limitations of purely clinical approaches to health, although systematic, practical solutions to address these social determinants of health have continued to lag far behind—until recently. 

Social prescribing offers a new approach. It connects patients with nonmedical resources in their communities, focussing on their individual priorities and measuring the impact on overall health and well-being.^2^ Critically, social prescribing embeds health promotion—the increased capacity for individuals and communities to take control over their health and its determinants^3^—into health systems by providing both social supports and a paradigm shift away from a deficit-based focus (i.e. “What’s the matter with us?”) to a holistic, strengths-based focus (i.e. “What matters to us?”).^4^ While these principles have always been important, they are particularly needed in the wake of the COVID-19 pandemic, which has significantly taxed health systems and health care providers.

Inspired in part by the growth of social prescribing in the United Kingdom and other countries,^5^ the practice of social prescribing is experiencing significant growth across Canada, with initiatives underway in every province.^6^ Some are focussed on particular populations, such as caregivers,^7^ older adults,^8^ Black communities,^9^ Indigenous populations,^10^ children and youth,^11^ and those living with mental health conditions.^12^ Some are focussed on specific interventions, such as food,^13^ nature,10 arts and culture,^14^ physical activity^15^ and social connection,^16^ or support mechanisms, such as community service databases and technologically enabled referrals.^16^-^18^

The papers in Part 1 of this special issue demonstrate that research and evaluation of social prescribing in Canada are growing alongside, and not far behind, this expansion in practice. Here, authors from across Canada—students, health professionals, researchers, community members, social services workers and more—explore the versatility and adaptability of social prescribing of different interventions by and for different populations, highlight the need for next-generation leadership by young people and those with community-based and lived expertise, and set out vital areas in need of further study.

Part 1 of this special issue includes several examples of social prescribing in action. Vaillancourt et al. explore how relationships with nature and connecting with the land are culturally based and can be beneficial for health for both Indigenous and non-Indigenous communities.^10^ Their reflexive practice highlights the importance of decolonization and incorporating Indigenous healing practices into social prescribing initiatives. Ramirez et al. explore how social prescribing can be adapted to build health equity for Black communities, focussing on how to create culturally safe programs built on trust and Afrocentric values.^19^ Finally, Brubacher et al. analyze a food-based social prescribing program in Guelph, Ontario.^20^ Their findings show the program not only improved key outcomes but also expanded access to social services, allowing participants to spend their limited resources on other essentials.

This issue also explores the need for further research on social prescribing. Ashe et al. review existing studies to identify how researchers currently measure social prescribing’s effectiveness and outcomes.^21^ While they find that mental and emotional well-being are well studied areas, more research is needed to understand the impact on physical health, thinking skills and memory. Additionally, the review emphasizes the importance of considering sociodemographic factors such as income, education and ethnicity when evaluating the program’s fairness and effectiveness for everyone. Little et al. focus their commentary on food prescribing in particular, exploring research needs and opportunities related to food at the intersections between health and social services, and individual and population-level actions for health.^22^

Because what matters to people and communities is at the heart of social prescribing, the expertise of participants with lived and living experience must also be at the heart of social prescribing research. We are fortunate to be able to include three powerful letters to the Editor in this issue. Norman,^23^ Barre^24^ and Paquette^25^ all share their lived experiences, highlighting the impact of social prescribing interventions and emphasizing the importance of participant expertise in both program development and health care professional training.

Highlighting the potential for social prescribing to become widely adopted by future health professionals, community leaders and researchers, Muhl et al. report on a surge in postsecondary student interest in social prescribing across the country.^26^ The authors call for students, health care systems and universities to work together to build partnerships and integrate social prescribing into teaching, research and everyday practice.

Social prescribing is a burgeoning field in Canada, and we received many excellent submissions for this issue. As a result, we plan to issue a second part to this issue in September 2024. We further encourage researchers, practitioners and funders to pursue research into social prescribing to ensure the growing practice is effective, equitable, meaningful, measurable and health promoting. Fifty years after the release of the Lalonde report on health promotion, social prescribing has become a potential cornerstone of health promotion and chronic disease prevention in Canada—perhaps for the next 50 years, or even more.

## Conflicts of interest

KM, KGC, and SA were Guest Editors for this issue of the HPCDP Journal, but removed themselves from the editorial decision-making associated with this manuscript.

## Statement

The content and views expressed in this article are those of the authors and do not necessarily reflect those of the Government of Canada.

## References

[B01] Lalonde M (1974). A new perspective on the health of Canadians. Government of Canada.

[B02] Muhl C, Mulligan K, Bayoumi I, Ashcroft R, Godfrey C Establishing internationally accepted conceptual and operational definitions of social prescribing through expert consensus: a Delphi study. BMJ Open.

[B03] Health promotion [Internet]. WHO.

[B04] What matters to you [Internet]. CISP.

[B05] Morse DF, Sandhu S, Mulligan K, et al (2022). Global developments in social prescribing. BMJ Glob Health.

[B06] Mulligan K, Hsiung S, Bloch G, et al, Healthc Q (2023). Social prescribing in Canada: a tool for integrating health and social care for underserved communities. Healthc Q.

[B07] (2024). A social prescription for care: new pan-Canadian social prescribing initiative aims to improve the well-being of caregivers across the country [Internet]. CCCE.

[B08] Rediscovering purpose, connection, and creativity in retirement years through social prescribing in British Columbia [Internet]. UWBC.

[B09] New Black-focused social prescribing project aims to improve health in Black communities with a proven holistic approach grounded in Afrocentric principles of wellbeing [Internet]. AHC.

[B10] Vaillancourt A, Barnstaple R, Robitaille N, Williams T Nature prescribing: emerging insights about reconciliation-based and culturally inclusive approaches from a tricultural community health centre. Health Promot Chronic Dis Prev Can.

[B11] Social prescribing [Internet]. Vanier Social Pediatric Hub.

[B12] Social prescribing for better mental health [Internet]. KDE Hub.

[B13] Little M, Dodd W, Grewal A, Stringer E, Richter A A 52-week fresh food prescribing program reduces food insecurity and improves fruit and vegetable consumption in Ontario, Canada. A 52-week fresh food prescribing program reduces food insecurity and improves fruit and vegetable consumption in Ontario, Canada. Research Square [Preprint].

[B14] Announcing arts on prescription pilot project [Internet]. BC Alliance for Arts and Culture.

[B15] Prescription to get active [Internet]. Prescription to Get Active.

[B16] Friendly calls program [Internet]. Canadian Red Cross.

[B17] Welcome to Clic Social [Internet]. Clic Social.

[B18] Just what the doctor ordered [Internet]. OSSU.

[B19] Ramirez S, Beaudin N, Rayner J, Price N, Townsend D Black-focused social prescribing: the importance of an Afrocentric approach. Health Promot Chronic Dis Prev Can.

[B20] Brubacher LJ, Little M, Richter A, Dodd W How does fresh food prescribing fit into the social service landscape. Health Promot Chronic Dis Prev Can.

[B21] Ashe MC, Santos IK, Alfares H, Chudyk AM, Esfandiari EE Outcomes and instruments used in social prescribing: a modified umbrella review. Health Promot Chronic Dis Prev Can.

[B22] Little M, Dodd W, Brubacher LJ, Richter A Food prescribing in Canada: evidence, critiques and opportunities. Food prescribing in Canada: evidence, critiques and opportunities. [Commentary.] Health Promot Chronic Dis Prev Can.

[B23] Norman M Nonclinical prescriptions gave me light of hope: perspectives from people with lived experiences. Nonclinical prescriptions gave me light of hope: perspectives from people with lived experiences. [Letter to the Editor.] Health Promot Chronic Dis Prev Can.

[B24] Barre S Patient voice at the core of social prescribing: perspectives from people with lived experiences. Patient voice at the core of social prescribing: perspectives from people with lived experiences. [Letter to the Editor.] Health Promot Chronic Dis Prev Can.

[B25] Paquette H Social prescribing training for doctors: perspectives from people with lived experiences. [Letter to the Editor.] Health Promot Chronic Dis Prev Can.

[B26] Muhl C, Ruhigisha M, McGarity-Shipley E Building the social prescribing student movement in Canada. Health Promot Chronic Dis Prev Can.

